# Pleural cavity cytokine release syndrome in CD19-directed chimeric antigen receptor-modified T cell therapy: A case report

**DOI:** 10.1097/MD.0000000000009992

**Published:** 2018-02-16

**Authors:** Lijuan Ding, Yongxian Hu, Kui Zhao, Guoqing Wei, Wenjun Wu, Zhao Wu, Lei Xiao, He Huang

**Affiliations:** a Bone Marrow Transplantation Center; b PET/CT Center, The First Affiliated Hospital, School of Medicine, Zhejiang University, Hangzhou; c Innovative Cellular Therapeutics Co., Ltd, Shanghai, China.

**Keywords:** CD19, chimeric antigen receptor-modified T cells, cytokine release syndrome, lymphoma, pleural cavity

## Abstract

**Rationale:**

Cytokine release syndrome (CRS) is a common and potentially fatal complication of CAR-T cell therapy. However, compartment CRS is relatively rare in hematological malignancies, as well as in solid tumors. The pathogenesis and prognosis of compartment CRS are unclear and there is no standardized treatment yet. In this case report, we will introduce a patient developing pleural cavity CRS after CART19s infusion.

**Patient concerns:**

A 28-year-old woman was admitted for evaluation of mediastinal mass. Her relevant examinations were comoleted.

**Diagnoses:**

She was diagnosed as diffuse large B cell lymphoma (DLBCL, non-GCB type).

**Interventions:**

She received chemotherapies including 1 cycle of R-DAEPORCH, 1 cycle of R-CHOPE, 2 cycles of R-CHOP, and 4 cycles of R-GDP during the disease course.

**Outcomes:**

The cytokine levels of hydrothorax were considerably high when serum cytokines were within normal range, with IL-6 at 1212.45 versus 5.69 pg/mL. qPCR analysis for CAR constructs showed 1,119,696 copies/μg DNA in hydrothorax and 522,227 copies/μg DNA in blood.

**Lessons:**

The results indicated that CART19 cells trafficked to the pleural cavity and interacted with the CD19-positive lymphoma cells directly, causing cytokine release in situ.

## 1 Introduction

Chimeric antigen receptor-modified T cells (CAR-T) have yielded unparalleled efficacy in B cell malignancies, most remarkably in CAR-T cells targeting CD19 (CART19s) for B cell acute lymphoblastic leukemia (B-ALL) with complete remission rate up to 93%.^[1–3]^ Results in non-Hodgkin lymphoma (NHL) have not been as striking as in ALL, with complete remission rate of 50% to 57%.^[4]^ However, cytokine release syndrome (CRS) has emerged as a potentially lethal complication. CRS is a systemic inflammatory reaction and characterized by the overwhelming release of cytokines, including IL-6, IL-10, IFN-γ, IL-2, IL-4, TNF-α, and IL-17.^[5]^ As a typical and high-incidence complication of CART cell therapy, CRS occurs within 1–10 days of infusion and generally lasts for several days up to 9 days.^[6]^ CRS spans the spectrum from mild to life threatening, but not all patients experience it. The syndrome typically begins within fever, with possibility to progress to hypotension, respiratory distress, capillary leak syndrome and neurological disorders such as headache, confusion, hallucination, and seizure. Steroids and tocilizumab have been used as the first-line agents in CRS. Tocilizumab, a monoclonal antibody binding IL-6 receptor, has been demonstrated to attenuate CRS toxicities, showing brilliant effect in fever, hypotension and respiratory distress.^[7]^ Lee et al^[8]^ divided CRS into 5 groups, and defined grade 3 CRS as the indicator of using glucocorticoids. Severe CRS refers to grade 4 to 5 CRS, which demands active and effective intervention interventions.

As lymphoma cells accumulate in the foci of lymphoma, the manifestation of CRS is characterized by local symptoms. So far, researches on CRS were mostly systemic CRS, while local CRS was rarely concerned. Here, we describe a patient developing pleural cavity CRS when undergoing autologous CART19 therapy.

## 2 Case presentation

A 28-year-old woman with intractable dry cough was delivered to a local hospital eight months ago. She was initially diagnosed with thymoma, however, positron emission tomography (PET)/computed tomography (CT) showed diffuse masses in the anterior mediastinum with multiple invasion to the lymph nodes around. Biopsy from cervical lymph node dissection confirmed the diagnosis of DLBCL (non-GCB). Immunohistochemistry analysis showed CD20 (+), CD5 (−), CD10 (−), BCL-2 (+), BCL-6 (+), EBV (−), Ki-67 (+, 80%), CyclinD1 (−). High expression level of Ki-67 revealed a highly invasive disease course. The patient underwent four chemotherapies including one course of R-DAEPORCH, 2 courses of R-CHOP, and 1 course of R-CHOPE. Following estimation revealed progressive disease course. Then the patient was started on 4 cycles of R-GDP. A repeat PET-CT after the last cycle showed no fadeaway of posterior mediastinal mass but additional breast mass. Then, the patient was recruited for CART19 clinical trial (ChiCTR-OCC-15007008) under a consent form. Right before the time of enrollment, an excisional biopsy on the left chest wall showed 14.14% of CD19-positive lymphoma cells. Peripheral-blood mononuclear cells (PBMCs) were collected before administration of FC regimen (Fludarabine 43 mg/m^2^ day 1 to 3, Cyclophosphamide 700 mg/m^2^ day 2 to 3). CART19s were generated as previously reported.^[9]^ The patient was infused with a total of 2.45 × 10^8^ CART19 cells (4.45 × 10^6^ CART19 cells per kilogram).

**Figure 1:**
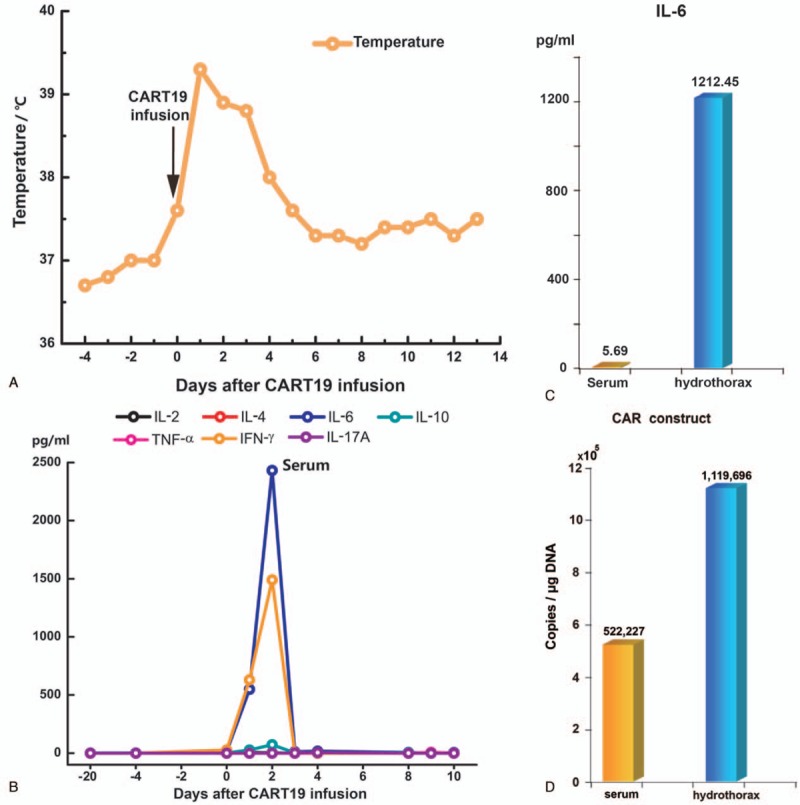
(A) Changes in body temperature after CART19 infusion, with a maximum temperature within 24 hours as indicated by the profile. (B) Cytokine levels in blood serum at different time points after CART19 infusion. (C) IL-6 level in peripheral serum and hydrothorax, respectively, on day 8. (D) DNA copies of CAR construct in blood serum and hydrothorax, respectively. CART19 = CD19-directed CAR-T cell.

**Figure 2:**
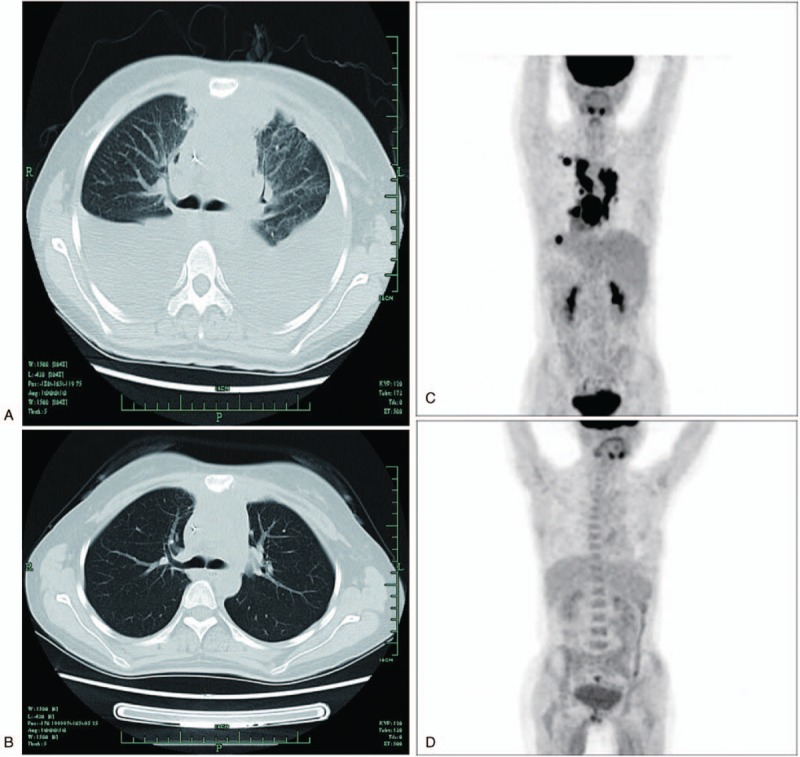
Changes of hydrothorax and masses of lymphoma infiltration after CART19 infusion. (A) Lung CT image on day 7 after CART19 infusion showing bilateral pleural effusion. (B) Lung CT image on day 21 after CART19 infusion showing that the pleural effusion on both sides were completely absorbed. (C) PET/CT before treatment showing high metabolic area in the chest. (D) The patient's chest was clean on day 28 after treatment. CART19 = CD19-directed CAR-T cell, CT = computed tomography, PET = positron emission tomography.

The next day after CART19s infusion, the patient got fever with a temperature of 39.3°C (102.7^ο^F). Figure 1A indicated body temperature changes on the time axis of 18 days. The levels of C-reactive protein (CRP) were increased beyond 100 mg/L. Multiple blood cultures were negative, which eliminated bacterial infection. The patient was suspected as CRS with distinct elevation of serum cytokines including INF-γ, IL-6, and IL-10, suggesting a grade 2 cytokine release syndrome (CRS) (Fig. 1B). According to the grading system by Lee's group, it was sufficient for disease control with anti-infection agents and supportive care. Three days later, the patient was suspected of pleural effusion for complaining of progressive cough, especially at lying position. CT scan showed bilateral pleural effusion (Fig. 2A and B). Further exploration with color Doppler ultrasound revealed 2 homogenous liquid dark area with widths of 5 to 6 cm (left) and 6 to 7 cm (right), respectively. We performed the thoracentesis. In contrast to the normal cytokine levels in the blood serum, the cytokine level of hydrothorax was considerably high, with IL-6 at 1212.45 versus 5.69 pg/mL (Fig. 1C). Meanwhile, qPCR analysis for CAR constructs showed 1,119,696 copies/μg DNA in hydrothorax versus 522,227 copies/μg DNA in blood (Fig. 1D). By day 9, all symptoms and signs attenuated, and blood test was almost normal. Reviewed CT scan indicated absorption of the pleural effusion on the left side. By day 21, the pleural effusion on both sides was completely absorbed. PET/CT on 1 month after CART19 infusion showed complete remission. (Fig. 2C and D). There was no sign of serious neurotoxicity or on-target off-tumor effect in the disease course.

## 3 Discussion

There have been several attempts to study compartment CRS. we reported for the first time a case of cerebral CRS following rapid remission after CART19 therapy in a relapsed/refractory ALL patient, which has not been reported before.^[9]^ Pleural cavity CRS has been reported as a rare case after treatment with mesothelin-targeted CAR-T cells.^[10]^ The patient in this case might be the first report of pleural cavity CRS referring to CART19 therapy.

The routine examination of pleural fluid confirmed that there was no increase of white blood cells or proteins, which eliminated pneumonia and pleuritic caused by infection. Tuberculosis was previously excluded. Considering the high cytokine levels in hydrothorax compared to in blood serum, we deduced that this patient had compartment CRS within the pleural cavity, which differed from systemic CRS. The presence of high levels CAR-T cells indicated that localized CRS in the pleural cavity could have been the effect of on-tumor rather than off-tumor targeting of CD19. Notably, the time of peak cytokine levels in hydrothorax emerged comparatively later than in blood, suggesting that pleural cavity and systemic CRS were independent processes.

The symptoms of pleural cavity CRS and systemic CRS can vary significantly. Systemic CRS typically begins within fevers with upregulated cytokines. In addition, patients may experience hypotension, respiratory distress, and neurologic disorders. In most hematological malignancies, tumor cells circle around where blood vessel extends, leading to bulk T cell activation. The consequences can be detected in secretion of large amounts of cytokine and activation of immune cells. Compartment CRS is confined in natural body cavity. CAR-T cells, activated by aggregated tumor cells, release a substantial amount of inflammatory cytokines in situ, which is how compartment CRS occurs. Accordingly, pleural cavity CRS is caused by CAR-T cells killing tumor cells in situ. The manifestation of pleural cavity CRS are mainly respiratory symptoms, such as cough, shortness of breath, chest tightness pain, as well as asphyxia. Severe pleural cavity CRS can be life-threatening, therefore, early recognition of pleural cavity CRS occupies important significance.
